# Tolerance to apical and leaf damage of *Raphanus raphanistrum* in different competitive regimes

**DOI:** 10.1002/ece3.1759

**Published:** 2015-10-22

**Authors:** Elin Dahlgren, Kari Lehtilä

**Affiliations:** ^1^ Legal Affairs Swedish Environmental Protection Agency SE‐10648 Stockholm Sweden; ^2^ The School of Natural Sciences, Technology and Environmental Studies Södertörn University SE‐14189 Huddinge Sweden

**Keywords:** Cost of tolerance, crucifers, herbivory, plant competition, trade‐off

## Abstract

Tolerance to herbivory is an adaptation that promotes regrowth and maintains fitness in plants after herbivore damage. Here, we hypothesized that the effect of competition on tolerance can be different for different genotypes within a species and we tested how tolerance is affected by competitive regime and damage type. We inflicted apical or leaf damage in siblings of 29 families of an annual plant *Raphanus raphanistrum* (Brassicaceae) grown at high or low competition. There was a negative correlation of family tolerance levels between competition treatments: plant families with high tolerance to apical damage in the low competition treatment had low tolerance to apical damage in the high competition treatment and vice versa. We found no costs of tolerance, in terms of a trade‐off between tolerance to apical and leaf damage or between tolerance and competitive ability, or an allocation cost in terms of reduced fitness of highly tolerant families in the undamaged state. High tolerance bound to a specific competitive regime may entail a cost in terms of low tolerance if competitive regime changes. This could act as a factor maintaining genetic variation for tolerance.

## Introduction

Defined as the ability to sustain tissue loss with little or no decrease in fitness after herbivore damage (Painter 1958), herbivory tolerance is, together with resistance and escape, one of the main types of plant adaptations to herbivory. Although stabilizing selection toward optimal tolerance could be expected for a trait closely linked to fitness, many plant populations often have substantial genetic variation for tolerance (Mauricio et al. 1997; Stowe 1998; Agrawal et al. 1999; Boalt and Lehtilä 2007). If tolerance is not favourable in all situations, negative side effects, costs of tolerance may in combination with environmental variation exert fluctuating selective pressures on tolerance and maintain genetic variation (Roff 1997). In the search of costs connected to tolerance, the most commonly tested type of tolerance cost is the resource allocation cost of maintaining tolerance mechanisms, resulting in reduced investments in other fitness‐related traits (Simms and Triplett 1994). High tolerance will in that case have a positive effect on fitness when herbivore pressure is strong, but affect fitness negatively in case of no herbivory. Evidence for this type of tolerance cost is not conclusive as some studies have observed such allocation cost (Tiffin and Rausher 1999), others have found the cost only in some of the reported experiments (Hochwender et al. 2000; Stinchcombe 2002; Fornoni et al. 2004), and some studies have failed to find the cost (Lennartsson et al. 1997; Agrawal et al. 1999; Fornoni and Núñez‐Farfán 2000; Juenger and Bergelson 2000; Boalt and Lehtilä 2007). Furthermore, it has been suggested that there is a cost in terms of a trade‐off between tolerance and resistance, if plant resources are limited and both defensive strategies have allocation costs (van der Meijden et al. 1988). Some studies have found evidence for this trade‐off (e.g., Fineblum and Rausher 1995, Stowe 1998). However, in a meta‐analysis, Leimu and Koricheva (2006) found no general support for a trade‐off between tolerance and resistance.

Herbivory tolerance may be associated with competitive ability. Hypotheses about the effect of environmental resource levels on tolerance are relevant in this case. The compensatory continuum hypothesis (Maschinski and Whitham 1989) predicts that tolerance is highest at high resource levels, whereas the growth rate model (Hilbert et al. 1981) claims that it is highest in stressful environments. Empirical studies have given partial support to both of these hypotheses, where much of the variation in results can be attributed to qualitative differences between monocot and dicot herbs (Hawkes and Sullivan 2001). In LRM (limiting resource model), the effect of environmental resource levels on tolerance is predicted by considering which factors limit plant fitness and which resources are affected by herbivory (Wise and Abrahamson 2005). According to the LRM, tolerance may either be relatively lower or higher in stressful conditions, depending on whether the resource affected by herbivores is the same resource that causes the environment to be stressful. Thus the effect of environmental resources on herbivory tolerance can depend on the type of herbivory. In its original form, LRM makes the generalization that the effect of resource levels on tolerance is similar among genotypes and populations within species (Banta et al. 2010). In this study, we analyse whether the effect of competition on tolerance varies among genotypes within a species and if it can affect selection pressures on tolerance. We are aware of only two studies testing how plant competition affects tolerance of different genotypes. Tiffin (2002) did not find any association between genotypic levels of tolerance to leaf damage in high and low competition in the morning glory *Ipomoea purpurea*. Similarly, Siemens et al. (2003) could not observe any association between tolerance levels of individuals of same families of *Arabis perennans* grown in high and low competition.

It is not known whether competitive regime has a similar or different effect on tolerance to apical and foliar herbivory. Most studies of tolerance are limited either to apical damage (Huhta et al. 2000; van der Meijden et al. 2000; Weinig et al. 2003; Rautio et al. 2005) or to foliar damage (Agrawal et al. 1999; Tiffin 2002; Siemens et al. 2003; Strauss et al. 2003). Studies that have simultaneously examined tolerance to foliar and apical damage have found either a positive genetic correlation between these two types of tolerance (Tiffin and Rausher 1999) or no correlation between them (Boalt and Lehtilä 2007). Apical dominance may be an important trait in this matter, because both competitive regime and damage type may affect the strength of apical dominance. When herbivore damage on apical parts disturbs the apical dominance, outcomes may vary: the damage may either trigger the growth of lateral branches, or apical dominance may be quickly restored resulting in height growth. If apical dominance is so strong before damage that meristem availability limits fitness, branching is an effective way to compensate for damage (Geber 1989; Aarssen 1995). This is more likely when light and other environmental resources are plentiful (Maschinski and Whitham 1989). On the other hand, if light competition is intense and branching reduces height growth, rapid restoration of apical dominance should be favourable for plant fitness. It is thus possible that the effectiveness of branching as a tolerance mechanism is dependent on competitive regime. If there is genetic variation in the tendency of branching after damage, it could lead to a trade‐off where certain genotypes are better developed to tolerate damage in certain competitive regimes. As apical dominance is strongly affected by apical damage, whereas foliar herbivory has only a weak or no effect on apical dominance, this type of trade‐off should be more probable for tolerance to apical than to foliar damage.

In this study, we examine the effects of competitive regime and different damage types on trade‐offs involved in tolerance. Under both high and low competitive regimes we tested (1) whether *Raphanus raphanistrum*, wild radish, shows the cost of tolerance in terms of low fitness of highly tolerant genotypes when herbivores are absent, and (2) whether there is a trade‐off between tolerance to different types of damage, or in high and low competitive regimes. When tolerance was found to differ between competitive regimes, we studied how the release of apical dominance was involved in the expression of tolerance. We analysed whether branching after damage was associated with tolerance in different competitive regimes and whether it was negatively correlated with height growth.

## Materials and Methods

### Study species

Wild radish, *Raphanus raphanistrum* L. (Brassicaceae), is a self‐incompatible annual plant (Sampson 1964), widely found in agricultural fields and disturbed areas. In Scandinavia, *R. raphanistrum* emerges during the spring and produces flowers continuously from July to September until plants die with the first frost. Fruits mature into elongated siliques, which are up to 9 cm long. *R. raphanistrum* plants are naturally visited by a large number of herbivores. Up to 50% foliar damage due to herbivory is not uncommon in the field (K. Lehtilä, unpubl. data). Naturally occurring herbivores observed in our field location were striped turnip flea beetles (*Phyllotreta nemorum*), pollen beetles (*Meligethes aenus*), large white butterflies (*Pieris brassicae*), and cabbage white butterflies (*Pieris rapae*). Herbivores known to cause apical damage on plants include rabbits, hares, and ungulates.

### Experimental design

Seeds from known maternal plants of *R. raphanistrum* were collected from the wild at Lieto in southwest Finland. The seeds were sown in the greenhouse to produce one offspring for each maternal plant as a parental generation of our experimental plants. The plants of the parental generation were randomly assigned as either paternal or maternal plants in crossings to produce full‐sib families with hand‐pollinations. Full sibs enable the exposure of close relatives to different damage treatments and reduce maternal effects. Different parent plants were used for each family. Parental plants of crossings that did not produce seed due to incompatibility were removed from the experiment.

The experimental setup was a factorial design with two blocks, two levels of competition (high and low), and three herbivory treatments (apical damage, leaf damage, and undamaged control). Each of the 29 plant families was replicated two times in treatment combinations that included apical and leaf herbivory, and four times in the treatment combinations with no herbivory (controls).

In the beginning of June 2004, *R. raphanistrum* plants that were to act as competitive, nonfocal plants were transplanted to a previously plowed and disked field in Gnesta, south‐eastern Sweden. Two weeks later, *R. raphanistrum* seeds from 29 full‐sib families that were to act as focal plants (i.e. plants from which measurements are recorded) were placed on moisturized filter paper in petri dishes to germinate. When the seedlings reached over five centimetres in length, they were transplanted to the field location. The experiment consisted of 464 plants of which 439 plants survived the experiment; 25 plants died directly after transplantation and were not included in the statistical analyses. Flowering resulted in seed set in 189 and 101 plants in the low and high competition treatments respectively.

Neighbouring, nonfocal plants surrounded the plants in the high competitive regime, where the distance to the four nearest neighbours was five centimetres. In the low competitive regime, the distance to the four nearest neighbours was 30 centimetres. To avoid edge effects, nonfocal plants were planted to create the desired competition effects in the end of rows. Within each competition treatment, eight plants from each of 29 families were randomly subjected to three experimental treatments, with two plants belonging to the apical damage treatment, two plants to the leaf damage treatment and four plants to the control group. In the leaf damage treatment, naturally occurring herbivores were allowed to free access to the plants. In order to standardize the amount of damage degree, supplemental clipping with scissors produced a 30% tissue loss to all plants. In no case did the naturally occurring herbivores inflict more than 30% damage and all plants in the leaf herbivore treatment were subjected to a combination of natural and artificial damage. To mimic apical herbivory on plants in the apical damage group, the first bolting shoot reaching 5 cm was removed using scissors. To minimize unwanted insect herbivory, naturally occurring herbivores, mainly butterfly larvae, were removed by hand on control and apical damaged plants. Control plants were also sprayed once every 7 days with insecticide Pyrenol, with pyrethrine as the active substance. In earlier studies, pyrethrine has not shown any effects on plant performance (Prittinen 2005). Despite the use of pesticides, control plants suffered minor (<1%) herbivore damage.

When studying plant responses to various forms of herbivory, simulated damage is often a useful tool in order to provide standardized amounts of damage. In a literature review (Lehtilä and Boalt 2004) we found that although artificial damage often has different effects than damage by real herbivores, the difference is more pronounced for response variables measuring chemical responses, compared to measurements of traits often involved in tolerance, such as growth and reproduction. Based on this, the outcomes of the combined effects of artificial and natural herbivory used in the present study is believed to have resulted in relevant responses in our study plants.

### Traits measured

Several vegetative and reproductive traits were measured to document the effect of damage and competition on plant performance. The first day of flowering was recorded for each plant. Width and length of two adjacent petals on one of the 20 first developed flowers were measured with a digital calliper. Petal size (mm^2^) was calculated as 0.712 × petal length × petal width (Boalt and Lehtilä 2007). The number of developed inflorescences was counted. In *R. raphanistrum*, the number of inflorescences is approximately the same as the number of branches, because all branches produce a terminal inflorescence. Total plant height was measured after seed harvest. As an estimate of leaf size, we measured length × width at the widest point of the left half of the largest leaf on each plant (this was possible even after leaf damage). Measurements of leaves were conducted at the time of flower initiation. The leaf size measure was taken from full‐grown leaves after the damage treatment and possible compensatory growth. Fruits were collected when they matured. All fruits and seeds were counted at the end of the experiment. The number of seeds serves as our fitness estimate. We did not estimate the male fitness of the experimental plants.

### Data analysis

The effects of apical and leaf damage on all plant traits except the number of seeds were analysed with linear mixed effects models using the lmer function of package lme4 (Bates et al. 2014) in R 3.1.2 (R Development Core Team 2014). The design had block as a main effect and all factorial combinations of family, damage treatment and competition treatment. We considered main family effect and all interactions including family as random effects, and block, damage and competition treatments as fixed effects. The area of the largest leaf and the number of inflorescences were log‐transformed. In case of plants failing to reproduce, mainly due to reduced size and delayed flowering, the number of seeds was assigned to zero. Similarly, the number of inflorescences was assigned to zero when plants did not produce flowering stems and flower, whereas the first day of flowering and petal area were assigned missing values because zero values are not reasonable for these traits. *χ* ^2^ values were calculated with likelihood ratio tests of models with and without the focal effect (and both models without higher‐order terms containing the effect under test), using restricted maximum likelihoods when testing random effects and maximum likelihoods when testing fixed effects (Faraway 2006). *P* values were calculated with parametric bootstrap of 1000 bootstrap replications (Faraway 2006). Dunnett's tests were conducted for pairwise comparisons of herbivory treatments with the control group. Normality of residuals was checked from normal probability plots and homoscedasticity from plots of fitted values versus residuals. We also checked that random effects were normally distributed by inspecting normal probability plots of each random effect versus residuals. Best linear unbiased predictors (BLUPs) derived from random effects were used when family estimates of plant traits were needed in analyses (Faraway 2006). Means and standard errors presented in the figures were calculated from model estimates.

Because the number of seeds was not normally distributed, it was tested with a generalized linear mixed effect model with negative binomial errors using glmmadmb function of the package glmmADMB in R (Skaug et al. 2011). A significant family × damage interaction on seed production would indicate genotypic variation among families in their response to leaf and/or apical damage, i.e., genotypic variation in herbivory tolerance. To make separate a posteriori tests of family × leaf damage and family × apical damage interactions, we removed the plants of either apical damage or leaf damage treatment from the analysis, carried out the tests and adjusted the *P* values of family × damage interaction with Dunn‐Šidák correction to control the experimentwise error rate (Day and Quinn 1989). *χ* ^2^ and *P* values were calculated with likelihood ratio tests of models with and without the focal effect. Likelihoods were estimated with Laplace approximation (Bolker et al. 2009). We did not use parametric bootstrap because simulations would have been very time consuming and because glmmADMB lacks simulate function that is important in producing simulated data for bootstrap estimates. Homogeneity of variance was checked from a plot of the predicted values versus residuals and by graphical comparison of the variances of the residuals across the families (Bolker et al. 2009).

Tolerance values of plant families were calculated as slopes of regression of seed production on damage level (Simms and Triplett 1994). Tolerance to leaf damage, referred here as leaf tolerance, was calculated as the slope of regression of seed production on damage in the control and leaf damage treatments within each family and competition treatment combination. Tolerance to apical damage, referred as apical tolerance, was the slope of regression in the control and apical damage treatments. Wise and Carr (2008) recommend to avoid combining additive and proportional scales in estimating tolerance. We used proportional scale, because the leaf damage treatment was carried out in proportional scale, and apical removal of the only stem of young plants can be interpreted as a damage treatment in either additive or proportional scale. In statistics, the generalized linear mixed effects model with the log link function uses proportional scale. BLUPs of the generalized mixed effects models were used to calculate the seed production and tolerance values of each family and experimental group (Carmona and Fornoni 2012). In figures, tolerances were back‐transformed but plotted in logarithmic axes to show the patterns in the same scale as in the statistical analyses. Back‐transformed tolerance values show the ratio of seed production between damage treatment (apical damage or 30% leaf removal) and control group. For instance, back‐transformed tolerance of 0.5 means that the average seed production of damage treatment was half of the average seed production of the control group.

When we tested for the association of tolerance with fitness of undamaged plants, we divided the four control individuals of each genotype into two separate groups to avoid autocorrelation. Half of the control group was used to calculate the tolerance and the other half to calculate independent estimates of trait values in zero damage. Similarly, when testing the relationship between tolerances to apical versus leaf damage, different control groups were used to calculate the two types of tolerance. We conducted two‐sided Pearson correlation tests of genotypic values of apical and leaf tolerance between the different competitive regimes. Spearman correlation was used in correlation tests with non‐normal test variables.

## Results

### Effects of competition and damage

**Figure 1:**
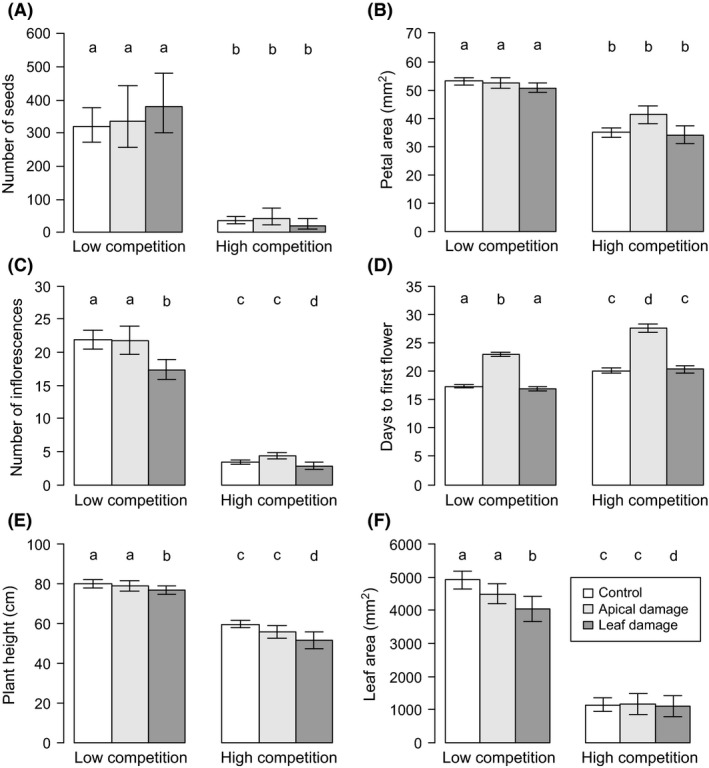
Effect of competition (horizontal axis labels) and apical and leaf damage on reproductive and vegetative traits of *Raphanus raphanistrum*. Mean ± SE of the mean derived from coefficients of generalized linear mixed model (number of seeds) or linear mixed model (the other traits). Different letters above bars indicate significant difference between experimental groups (Dunnett's test between herbivory treatments and control group).

The high competition treatment resulted in a significantly reduced performance in all measured plant traits (Tables 1 and 2). In the high competition treatment, seed production decreased by 91%, petal area by 29%, and number of inflorescences by 83% compared to the low competition treatment (Fig. 1A–C). Days to first flower increased from 19 in low competition to 23 in high competition (Fig. 1D). Plant height and leaf area were reduced by 29% and 75%, respectively, from low to high competition (Fig. 1E,F).

**Table 1:** Effect of competition and damage on floral and vegetative traits of *Raphanus raphanistrum*. Mixed effects models with family and interactions with family as random effects and the other factors as fixed effects. *χ* ^2^ and degrees of freedoms (subscripts) from likelihood ratio tests with restricted maximum likelihoods for random effects and maximum likelihoods for fixed effects. *P* values were calculated with parametric bootstrap

	Block		Family		Damage		Competition		Dam×Comp		Fam×Dam		Fam×Comp		Fam×Dam×Comp	
	*χ* ^2^_df_	*P*	*χ* ^2^_df_	*P*	*χ* ^2^_df_	*P*	*χ* ^2^_df_	*P*	*χ* ^2^_df_	*P*	*χ* ^2^_df_	*P*	*χ* ^2^_df_	*P*	*χ* ^2^_df_	*P*
Petal area	1.00_1_	0.321	9.77_1_	0.001	3.79_2_	0.152	117_1_	<0.001	4.54_2_	0.134	3.01_7_	0.635	2.83_4_	0.273	1.69_11_	0.942
Nr of inflorescences	9.96_1_	0.002	0.004_1_	0.416	11.0_2_	0.005	352_1_	<0.001	2.63_2_	0.290	5.42_7_	0.237	3.10_2_	0.078	4.69_11_	0.517
Days to first flower	0.79_1_	0.375	11.8_1_	<0.001	211_2_	<0.001	100_1_	<0.001	5.14_2_	0.083	10.8_7_	0.028	10.6_4_	0.005	10.2_11_	0.121
Plant height	8.77_1_	0.003	2.71_1_	0.034	5.83_2_	0.047	135_1_	<0.001	1.57_2_	0.479	7.42_7_	0.105	2.44_4_	0.332	9.05_11_	0.190
Area of largest leaf	7.09_1_	0.010	11.4_1_	0.001	10.1_2_	0.007	316_1_	<0.001	1.56_2_	0.459	8.56_7_	0.091	2.28_2_	0.175	7.99_11_	0.250

**Table 2:** Effect of competition and herbivore damage on seed set of *Raphanus raphanistrum*. Generalized linear mixed model with negative binomial errors. Family and interactions with family as random effects and the other factors as fixed effects. *χ* ^2^ was calculated by likelihood ratio tests with Laplace approximation

Source	*χ* ^2^	df	*P*
Block	22.3	1	<0.001
Family	45.9	1	<0.001
Damage	0.68	2	0.712
Competition	340	1	<0.001
Competition × damage	3.68	2	0.159
Competition × family	4.22	1	0.040
Family × damage	8.78	2	0.012
Competition × family × damage	5.28	2	0.071

Leaf damage decreased the number of inflorescences with 20% compared to controls (Fig. 1C). Apical damage resulted in significantly delayed flowering with an average of six and a half days compared to controls (Table 1, Fig. 1D). Leaf damage decreased the plant height with 8% and the area of largest leaf by 15% compared to controls (Table 1, Fig. 1E,F).

### Genotypic variation of tolerance

**Figure 2:**
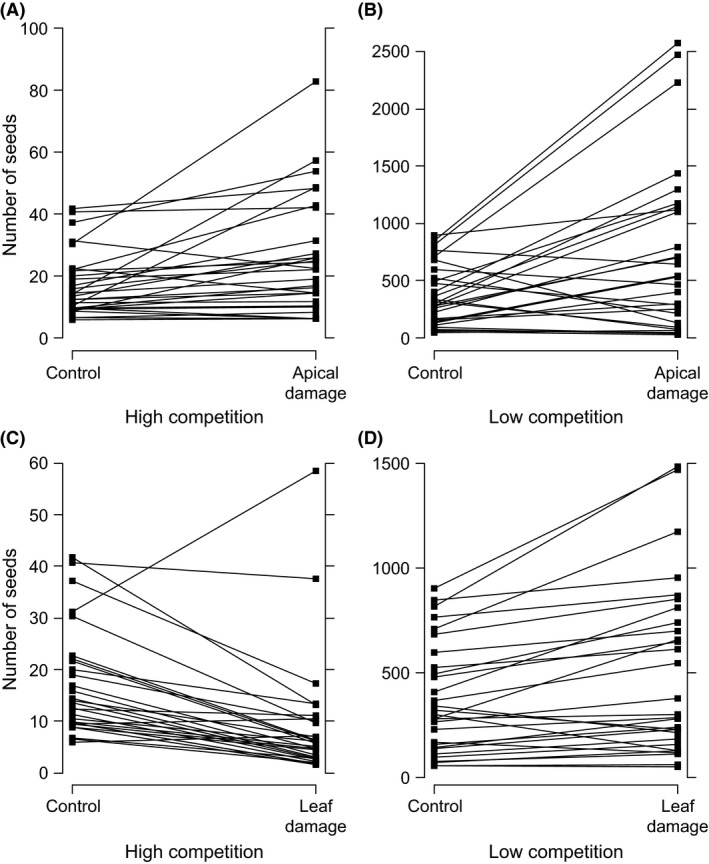
Scatterplot of apical tolerance levels of families grown in low and high competition. Apical tolerance is the slope of regression between level of apical damage and number of seeds. Tolerance values back‐transformed from logarithmic scale, showing family‐wise ratio between average seed numbers of apical damage and control group. Pearson's *r *=* *−0.445, *P *=* *0.020.

There was a significant genotypic variation of tolerance, as indicated by significant interaction between family and damage treatment (Table 2, Fig. 2). We carried out a posteriori analyses to test the significance of genotypic variation of apical and leaf tolerance. Family × apical damage interaction was significant but family × leaf damage interaction was not (family × apical damage, *χ* ^2^ = 12.7, df = 1, *P *<* *0.001; family × leaf damage, *χ* ^2^ = 0.30, df = 1, *P *=* *0.827; Dunn‐Šidák‐corrected *P* values for multiple comparisons).

### Trade‐offs involved in tolerance

We found no evidence for resource allocation costs involved with tolerance. Tolerance was not significantly negatively correlated with seed production of an independent (undamaged) control group in the low competition treatment (apical tolerance, *r* = 0.099, *P *=* *0.609, *N *=* *29; leaf tolerance, *r *=* *0.094, *P *=* *0.627, *N *=* *29). In the high competition treatment, leaf tolerance was positively correlated with seed production of an independent control group (apical tolerance, *r *=* *−0.266, *P *=* *0.180, *N *=* *27; leaf tolerance, *r *=* *0.807, *P *<* *0.001, *N *=* *28). We did not find any genetic trade‐off between tolerance to leaf and apical damage, as tolerances to the different damage types were not significantly correlated (low competition, *r* = 0.219, *P *=* *0.253, *N *=* *29; high competition, *r *=* *−0.093, *P *=* *0.652, *N *=* *26).

**Figure 3:**
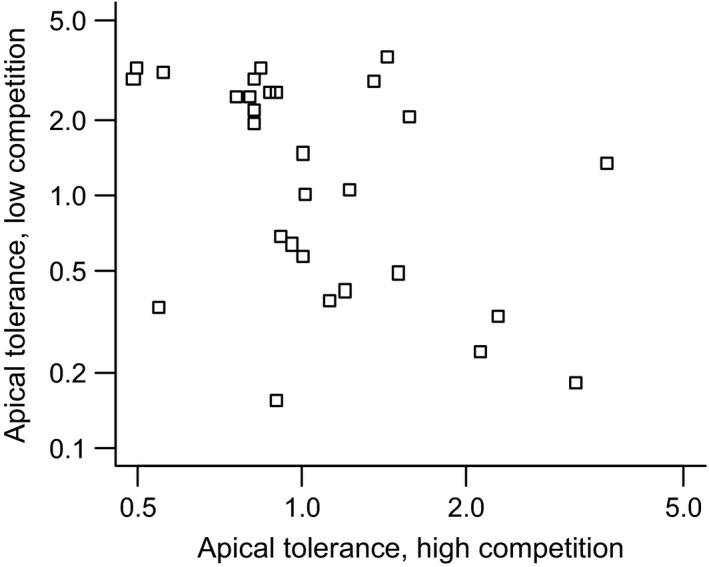
Herbivory tolerance of *Raphanus raphanistrum* to apical and leaf damage in high and low competition as reaction norms. Seed number of full‐sib families derived from coefficients of generalized linear mixed model. Increasing slopes indicate higher tolerance.

A trade‐off between tolerance to apical damage in different competitive regimes was observed as a significant negative correlation between the levels of apical tolerance in the high and low competitive treatments (*r *=* *−0.445, *P *=* *0.020, *N *=* *27, Fig. 3). For leaf tolerance, no such trade‐off was detected as levels of tolerance were not significantly correlated between competitive treatment (*r *=* *−0.031, *P *=* *0.875, *N *=* *28).

We tested whether branching responses after apical damage could explain why the relative genotypic levels of apical tolerance differed between the competitive regimes. Difference between apical damage and control treatments in the genotypic average of inflorescence number was used as an indicator of branching response to damage (all branches produce an inflorescence in *R. raphanistrum*). Positive correlation with apical tolerance and strong branching response after apical damage was significant in low competition and marginally significant in high competition (low competition, *r *=* *0.518, *P *=* *0.007, *N *=* *26; high competition, *r *=* *0.359, *P *<* *0.093, *N *=* *23). There was a significant negative correlation between branching responses after apical damage in high and low competition (*r *=* *−0.573, *P *=* *0.008, *N *=* *20), corresponding to the negative correlation of apical tolerance in high and low competition. These results suggest that branching after apical damage is an important tolerance mechanism, with the caveat that we did not observe any genotypic variation in inflorescence number (family effect and its interactions, Table 1). We did not observe any trade‐off between branching and height growth after apical damage. On the contrary, genotypes with strong branching response also had good ability for compensatory height growth after damage. The ability for height growth after apical damage, i.e. the difference in plant height between apical damage and control treatments, was positively correlated with branching response in both competition treatments (high competition, *r *=* *0.633, *P *=* *0.001, *H *=* *23; low competition, *r *=* *0.418, *P *=* *0.034, *N *=* *26). To analyse the patterns of phenotypic selection on inflorescence number and plant height (Lande and Arnold 1983), we added their standardized values as covariates to the model of Table 2. Selection among models including different interaction terms of the covariates and the competition and damage treatments was carried out with Akaike information criterion. Because the logarithmic link function could complicate the interpretation of partial regression coefficients (Morrissey and Sakredja 2013), we graphically checked that the patterns were similar in logarithmic and back‐transformed scale. The final model showed that both the number of inflorescences and height had a significant positive effect on the seed number and that the effect was stronger for the number of inflorescences than for height in their observed ranges (inflorescence number, *β *= 1.15, *P* < 0.001; height, *β *= 0.3102, *P *<* *0.001). There was a significant interaction between inflorescence number and competition treatment (*γ* = −0.955, *P *<* *0.001). Inflorescence number had a positive effect on seed number in both competition treatments, but the slope was steeper in low competition. Interaction between height and competition treatment was dropped from the final model. There was also a significant interaction between inflorescence number and height (*γ* = −0.155, *P *<* *0.001). Within the observed ranges of the covariates, the regression slope of each covariate was strongly positive with low values of the other covariate and weakly positive when the other covariate had a high value.

## Discussion

We found that there was a negative relationship among families of *R. raphanistrum* in tolerance to apical damage between high and low competition. High levels of apical tolerance in high competition may thus come at a cost of low levels of apical tolerance in low competition, and vice versa. Similarly as Tiffin (2002) and Siemens et al. (2003), we found no significant correlation between the levels of tolerance to foliar damage in different competitive regimes. Previous studies have found differences in tolerance between populations exposed to different intensities in herbivory (Lennartsson et al. 1997; Boalt et al. 2010, Martin et al. 2015). As *R. raphanistrum* is an annual weed of ruderal habitats, most populations probably experience competitive regimes that vary among years. Genotypes having the best apical tolerance may vary according to competitive regime and thus maintain genotypic variation in tolerance.

We tested whether genotypic differences in branching after damage could explain the findings. Many studies have shown the importance of increased branch and inflorescence production for compensatory regrowth and tolerance (Inouye 1982; Paige and Whitham 1987; Benner 1988; Lennartsson et al. 1998). In *R. raphanistrum*, high branch number is associated with high inflorescence number, because branches always produce an inflorescence. We predicted that high propensity of branching would be beneficial for tolerance in low competition but detrimental in high competition. This assumes that there is a trade‐off between branching and height growth, and that height growth is more important for light capture in high than in low competition. However, there was no trade‐off between branching and height growth, but they were positively correlated. We did not observe any negative relationship between apical tolerance and branching after apical damage in either high or low competition. Height and inflorescence number may both be indicators of an overall large size. Phenotypic selection was stronger on inflorescence number than on height, and it was positive for both traits in both competition treatments, although for high inflorescence number selection was stronger in low than in high competition.

In order to understand the evolution and maintenance of genetic variation of tolerance, we investigated the presence of a direct fitness cost and indirect costs in terms of trade‐offs. Some studies have shown that there are allocation costs of tolerance, that is, reduced fitness of highly tolerant genotypes in the absence of herbivory (Tiffin and Rausher 1999; Hochwender et al. 2000; Stinchcombe 2002; Fornoni et al. 2004). However, we found no evidence of a direct fitness cost of tolerance, similarly as many other studies (Lennartsson et al. 1997; Mauricio et al. 1997; Agrawal et al. 1999; Juenger and Bergelson 2000; Boalt and Lehtilä 2007). A cost of tolerance as a negative correlation between apical and leaf tolerance has been shown in the annual plant *Ipomoea purpurea* (Tiffin and Rausher 1999). In the present study and in an earlier study with *R. raphanistrum* (Boalt and Lehtilä 2007), we found no correlation between apical and leaf tolerance. Low statistical power must be taken into account when interpreting these results. For instance, at least one hundred families are needed to test a correlation of −0.25 with a high power (power = 0.8, *P* = 0.05). Few studies have had sufficient sample sizes to have a high power in tests of moderate, but still biologically important, costs of tolerance.

Wise and Abrahamson (2005) presented the LRM of the effects of environmental resource levels on herbivory tolerance, that integrates the earlier models of Hilbert et al. (1981) and Maschinski and Whitham (1989) into a comprehensive framework. The model predicts the plant tolerance based on resource limitation and resource use in different environments. If we try to use our results to test LRM or the other models, an important assumption is revealed that complicates the interpretations: the models, in their original form, give only one prediction for each species and a specific resource condition. We observed, however, that there was genetic variation in the effect of resource levels on herbivory tolerance. Some genotypes had a greater tolerance in high competition than in low competition, whereas in other genotypes the relationship was the opposite. Different genotypes from the same population thus show different outcomes. In line with other studies suggesting that models of herbivory tolerance would benefit of including more specific information on herbivore‐mediated transitions between water and nutrient limitation (Marshall et al. 2008), or on plasticity of resource uptake and allocation (Bagchi and Ritchie 2011), our results indicate that models would benefit of including more specific information on ecological and ecophysiological factors affecting plant responses to herbivores.

In summary, our findings suggest that plant competition is important for the expression of tolerance, as there was a negative correlation in genotypic levels of apical tolerance between the different competitive regimes. Because *R. raphanistrum* is commonly found in disturbed habitats that may be rapidly overgrown, competitive regime is highly variable. We suggest that a combination of varying competitive regimes and levels of herbivory may prevent the fixation of tolerance at a single optimal level. The findings also show that the effect of resource manipulations on tolerance can vary within a population, which does not conform with assumptions of general models of herbivory tolerance.

## Conflict of Interest

None declared.

## References

[ece31759-bib-0001] Aarssen, L. W. 1995 Hypotheses for the evolution of apical dominance in plants: implications for the interpretation of overcompensation. Oikos 74:149–156.

[ece31759-bib-0002] Agrawal, A. A. , S. Strauss , and M. J. Stout . 1999 Costs of induced responses and tolerance to herbivory in male and female fitness components of wild radish. Evolution 53:1093–1104. 28565524 10.1111/j.1558-5646.1999.tb04524.x

[ece31759-bib-0003] Bagchi, S. , and M. E. Ritchie . 2011 Herbivory and plant tolerance: experimental tests of alternative hypotheses involving non‐substitutable resources. Oikos 120:119–127.

[ece31759-bib-0004] Banta, J. A. , M. H. H. Stevens , and M. Pigliucci . 2010 A comprehensive test of the ‘limiting resources’ framework applied to plant tolerance to apical meristem damage. Oikos 119:359–369.

[ece31759-bib-0005] Bates, D. , M. Maechler , B. M. Bolker , and S. Walker . 2014 lme4: linear mixed effects models using Eigen and S4. R package version 1.1‐7. R Foundation for Statistical Computing, Vienna, Austria Available at http://cran.r-project.org/web/packages/lme4/ (accessed 30 April 2015).

[ece31759-bib-0006] Benner, B. 1988 Effects of apex removal and nutrient supplementation on branching and seed production in *Thlaspi arvense* (Brassicaceae). Am. J. Bot. 75:645–651. 10.1002/j.1537-2197.1988.tb13487.x 30139088

[ece31759-bib-0007] Boalt, E. , and K. Lehtilä . 2007 Tolerance to apical and foliar damage: costs and mechanisms in *Raphanus raphanistrum* . Oikos 116:2071–2081.

[ece31759-bib-0505] Boalt, E. , L. Arvanitis , K. Lehtilä , and J. Ehrlén . 2010 The association among herbivory tolerance, ploidy level, and herbivory pressure in *Cardamine pratensis* . Evol. Ecol. 24:1101–1113.

[ece31759-bib-0008] Bolker, B. M. , M. E. Brooks , C. J. Clark , S. W. Geange , J. R. Poulsen , M. H. H. Stevens , et al. 2009 Generalized linear mixed models: a practical guide for ecology and evolution. Trends Ecol. Evol. 24:127–135. 19185386 10.1016/j.tree.2008.10.008

[ece31759-bib-0009] Carmona, D. , and J. Fornoni . 2012 Herbivores can select for mixed defensive strategies in plants. New Phytol. 197:576–585. 23171270 10.1111/nph.12023

[ece31759-bib-0011] Day, R. W. , and G. P. Quinn . 1989 Comparisons of treatments after an analysis of variance in ecology. Ecol. Monogr. 59:433–463.

[ece31759-bib-0012] Faraway, J. J. 2006 Extending the linear model with R. Taylor and Francis, Boca Raton.

[ece31759-bib-0506] Fineblum, W. L. , and M. D. Rausher . 1995 Tradeoff between resistance and tolerance to herbivore damage in a morning glory. Nature 377:517–520.

[ece31759-bib-0013] Fornoni, J. , and J. Núñez‐Farfán . 2000 Evolutionary ecology of *Datura stramonium*: genetic variation and costs for tolerance to defoliation. Evolution 54:789–797. 10937253

[ece31759-bib-0014] Fornoni, J. , P. L. Valverde , and J. Nunez‐Farfan . 2004 Population variation in the cost and benefit of tolerance and resistance against herbivory in *Datura stramonium* . Evolution 58:1696–1704. 15446424

[ece31759-bib-0016] Geber, M. A. 1989 Interplay of morphology and development on size inequality: a *Polygonum* greenhouse study. Ecol. Monogr. 59:267–288.

[ece31759-bib-0018] Hawkes, C. V. , and J. J. Sullivan . 2001 The impact of herbivory in different resource conditions: a meta‐analysis. Ecology 82:2045–2058.

[ece31759-bib-0019] Hilbert, D. W. , D. M. Swift , J. K. Detling , and M. I. Dyer . 1981 Relative growth rates and the grazing optimization hypothesis. Oecologia 51:14–18. 28310302 10.1007/BF00344645

[ece31759-bib-0020] Hochwender, C. G. , R. J. Marquis , and K. A. Stowe . 2000 The potential for and constraints on the evolution of compensatory ability in *Asclepias syriaca* . Oecologia 122:361–370. 28308287 10.1007/s004420050042

[ece31759-bib-0021] Huhta, A.‐P. , K. Hellström , P. Rautio , and J. Tuomi . 2000 A test of the compensatory continuum: fertilization increases and below‐ground competition decreases the grazing tolerance of tall wormseed mustard (*Erysimum strictum*). Evol. Ecol. 14:353–372.

[ece31759-bib-0022] Inouye, D. W. 1982 The consequences of herbivory: a mixed blessing *for Jurinea mollis* (Asteraceae). Oikos 39:269–272.

[ece31759-bib-0023] Juenger, T. , and J. Bergelson . 2000 The evolution of compensation to herbivory in scarlet gilia, *Ipomopsis aggregata*: herbivore‐imposed natural selection and the quantitative genetics of tolerance. Evolution 54:764–777. 10937251 10.1111/j.0014-3820.2000.tb00078.x

[ece31759-bib-0024] Lande, R. , and S. J. Arnold . 1983 The measurement of selection on correlated characters. Evolution 37:1210–1226. 28556011 10.1111/j.1558-5646.1983.tb00236.x

[ece31759-bib-0507] Lehtilä, K. , and E. Boalt . 2004 The use and usefulness of artificial herbivory in plant‐herbivore studies. Pp. 257–275 *in* Weisser W. W. and Siemann E., eds. Insects and ecosystem function. Springer, Berlin.

[ece31759-bib-0508] Leimu, R. , and J. Koricheva . 2006 A meta‐analysis of tradeoffs between plant tolerance and resistance to herbivores: combining the evidence from ecological and agricultural studies. Oikos 112:1–9.

[ece31759-bib-0025] Lennartsson, T. , J. Tuomi , and P. Nilsson . 1997 Evidence for an evolutionary history of overcompensation in the grassland biennial *Gentianella campestris* (Gentianaceae). Am. Nat. 79:1061–1072. 10.1086/286043 18811268

[ece31759-bib-0026] Lennartsson, T. , P. Nilsson , and J. Tuomi . 1998 Induction of overcompensation in the field gentian, *Gentianella campestris* . Ecology 79:1061–1072.

[ece31759-bib-0028] Marshall, C. B. , G. Avila‐Sakar , and E. G. Reekie . 2008 Effects of nutrient and CO_2_ availability on tolerance to herbivory in *Brassica rapa* . Plant Ecol. 196:1–13.

[ece31759-bib-0029] Martin, L. J. , A. Agrawal , and C. E. Kraft . 2015 Historically browsed jewelweed populations exhibit greater tolerance to deer herbivory than historically protected populations. J. Ecol. 103:243–249.

[ece31759-bib-0030] Maschinski, J. , and T. G. Whitham . 1989 The continuum of plant responses to herbivory: the influence of plant association, nutrient availability, and timing. Am. Nat. 134:1–19.

[ece31759-bib-0031] Mauricio, R. , M. D. Rausher , and D. S. Burdick . 1997 Variation in the defence strategies of plants: are resistance and tolerance mutually exclusive? Ecology 78:1301–1311.

[ece31759-bib-0050] van der Meijden, E. , N. J. De Boer , and C. A. M. Van Der Veen‐Van Wijk . 2000 Pattern of storage and regrowth in ragwort. Evol. Ecol. 14:439–455.

[ece31759-bib-0509] van der Meijden, E. , M. Wijn , and H. J. Verkaar . 1988 Defence and regrowth, alternative strategies in the struggle against herbivores. Oikos 51:355–363.

[ece31759-bib-0032] Morrissey, M. B. , and K. Sakredja . 2013 Unification of regression‐based methods for the analysis of natural selection. Evolution 67:2094–2100. 23815662 10.1111/evo.12077

[ece31759-bib-0034] Paige, K. N. , and T. G. Whitham . 1987 Overcompensation in response to mammalian herbivory: the advantage of being eaten. Am. Nat. 129:407–416.

[ece31759-bib-0035] Painter, R. 1958 Resistance of plants to insects. Annu. Rev. Entomol. 3:367–390.

[ece31759-bib-0036] Prittinen, K. 2005 Herbivory among competing seedlings: effects on silver birch populations. PhD dissertations in Biology No 35, University of Joensuu, Joensuu.

[ece31759-bib-0037] R Development Core Team . 2014 R: a language and environment for statistical computing. R foundation for Statistical Computing, Vienna, Austria Available at: http://www.R-project.org (accessed 30 April 2015).

[ece31759-bib-0038] Rautio, P. , A.‐P. Huhta , S. Piippo , J. Tuomi , T. Juenger , M. Saari , et al. 2005 Overcompensation and adaptive plasticity of apical damage in *Erysimum strictum* . Oikos 111:179–191.

[ece31759-bib-0039] Roff, D. A. 1997 Evolutionary quantitative genetics. Chapman and Hall, New York.

[ece31759-bib-0040] Sampson, D. R. 1964 A one‐locus self‐incompatibility system in *Raphanus raphanistrum* . Can. J. Genet. Cytol. 6:435–445.

[ece31759-bib-0041] Siemens, D. H. , H. Lischke , N. Maggiulli , S. Schürch , and B. A. Roy . 2003 Cost of resistance and tolerance under competition: the defense‐stress benefit hypothesis. Evol. Ecol. 17:247–263.

[ece31759-bib-0042] Simms, E. L. , and J. Triplett . 1994 Costs and benefits of plant responses to disease: resistance and tolerance. Evolution 48:1973–1985. 28565152 10.1111/j.1558-5646.1994.tb02227.x

[ece31759-bib-0043] Skaug, H. , D. Fournier , A. Nielsen , A. Magnusson , and B. M. Bolker . 2011 glmmADMB: Generalized Linear Mixed Models using AD Model Builder. R package version 0.7. Available at http://glmmadmb.r-forge.r-project.org (accessed 30 April 2015).

[ece31759-bib-0044] Stinchcombe, J. R. 2002 Environmental dependency in the expression of costs of tolerance to deer herbivory. Evolution 56:1063–1067. 12093020 10.1111/j.0014-3820.2002.tb01417.x

[ece31759-bib-0045] Stowe, K. A. 1998 Experimental evolution of resistance in *Brassica rapa*: correlated response of tolerance in lines selected for glucosinolate content. Evolution 52:703–712. 28565253 10.1111/j.1558-5646.1998.tb03695.x

[ece31759-bib-0047] Strauss, S. Y. , W. Watson , and M. Allen . 2003 Predictors of male and female tolerance to insect herbivory in *Raphanus raphanistrum* . Ecology 84:2074–2082.

[ece31759-bib-0048] Tiffin, P. 2002 Competition and time of damage affect the pattern of selection acting on plant defense against herbivores. Ecology 83:1981–1990.

[ece31759-bib-0049] Tiffin, P. , and M. D. Rausher . 1999 Genetic constraints and selection acting on tolerance to herbivory in the common morning glory *Ipomoea purpurea* . Am. Nat. 154:700–716. 10600614 10.1086/303271

[ece31759-bib-0052] Weinig, C. , J. R. Stinchcombe , and J. Schmitt . 2003 Evolutionary genetics of resistance and tolerance to natural herbivory in *Arabidopsis thaliana* . Evolution 57:1270–1280. 12894935 10.1111/j.0014-3820.2003.tb00335.x

[ece31759-bib-0054] Wise, M. J. , and W. G. Abrahamson . 2005 Beyond the compensatory continuum: environmental resource levels and plant tolerance to herbivory. Oikos 109:417–428.

[ece31759-bib-0055] Wise, M. J. , and D. E. Carr . 2008 On quantifying tolerance of herbivory for comparative analyses. Evolution 62:2429–2434. 18637836 10.1111/j.1558-5646.2008.00458.x

